# Supplementary material for: Sympathomimetic-Induced Hyperthermia and Hyponatremia: A Simulation Case for Emergency Medicine Residents
Source: MedEdPORTAL. 2021 Jan 29;17:11092. doi: 10.15766/mep_2374-8265.11092 (PMC7845472; doi:10.15766/mep_2374-8265.11092)
Supplement: Supplementary file 1 — Simulation Case Template.docxAlternate Simulation Case Template.docxEquipment List.docxLaboratory Results.docxBody Bag Cue Card.docxResident Questionnaire.docxCritical Action Checklist.docxBackground Info for Debrief.docx [file mep_2374-8265.11092-s001.zip › A. Simulation Case Template.docx]

| **Appendix A: MedEdPORTAL Simulation Case Template**  **SIMULATION CASE TITLE: Sympathomimetic Induced Hyperthermia and Hyponatremia: A Simulation Case for Emergency Medicine Residents**  AUTHORS: Ashlea Winfield, MD, MSPH^1^ , Elizabeth Black, MD^2^ , Michelle Sergel, MD^3,4^  ^1^Simulation Fellow, Department of Emergency Medicine, Cook County Health  ^2^ Toxicology Fellow, Department of Emergency Medicine, Cook County Health  ^3^ Director of Cook County Simulation Center, Department of Emergency Medicine, Cook County Health  ^4^ Co-Executive Director Rush Center for Clinical Skills and Simulation  **LEARNER AUDIENCE: Emergency Medicine Residents** | |
| --- | --- |
| **PATIENT NAME: Alexander Martel**  **PATIENT AGE: 23**  **CHIEF COMPLAINT: Altered Mental Status**  **PHYSICAL SETTING: Community Emergency Department** | |
|  | |
| **Brief narrative description of case** | *Mr. Martel arrives via Emergency Medical Services (EMS) for altered mental status. Club security guards noted the patient was confused and EMS was called. He is acutely intoxicated from 3,4-Methyl​enedioxy​methamphetamine (MDMA). As a result of this ingestion the patient will be exhibit symptoms commonly seen in sympathomimetic toxidromes such as agitation, hyperthermia, and hyponatremia. The patient should be promptly cooled but will not adequately respond until whole body packing is performed. After appropriate cooling the patient will have a seizure secondary to hyponatremia. Once hypertonic saline or sodium bicarbonate is given the seizure activity will resolve and the patient should be dispositioned to the ICU.* |
| **Primary Learning Objectives** | 1. *Demonstrate a linear approach to evaluating the patient with altered mental status* 2. *Identify hyperthermia and the underlying toxidrome utilizing physical exam and history* 3. *Initiate cooling methods for hyperthermia: cooled IV fluids, ice pack placement, methods of evaporative cooling such as fan + tepid water mist or sponge, and whole body packing* 4. *Manage seizure activity due to MDMA induced hyponatremia with hypertonic saline or sodium bicarbonate* |
| **Critical Actions** | 1. *Obtain vital signs including a core temperature* 2. *Obtain stat glucose in patient with altered mental status* 3. *Perform brief history and physical* 4. *Verbalize diagnosis as sympathomimetic toxidrome* 5. *Send CBC, CMP, and creatine kinase (CK)* 6. *Initiate cooling with cold IV fluids, evaporative (mist+fan or sponge+fan or wet blanket+fan) cooling, and ice packs* 7. *Recognize failure to adequately respond to conservative cooling measures and progress to whole body packing* 8. *Discontinue cooling methods when the patient is appropriately cooled to approximately 102F to prevent overshoot hypothermia* 9. *Manage hyponatremic seizure with hypertonic saline bolus or sodium bicarbonate* 10. *Obtain a CT Brain for a patient with acute altered mental status and new onset seizure activity* 11. *Contact poison control* 12. *Disposition patient to Medical Intensive Care Unit* |
| **Learner Preparation or Prework** | *No information should be given prior to the case as this would alert learners to the content of the clinical scenario* |

| Initial Presentation | | | |
| --- | --- | --- | --- |
| **Initial vital signs** | Temp – 39.1C (oral) 40.5 (rectal), HR – 155, RR – 20, BP - 105/66, SpO2 – 100% | | |
| **Overall Setting and Appearance** | *The patient will be mumbling incoherently in stretcher, occasional swatting if touched, but not overtly violent. The patient will occasionally open his eyes and look around, occasional bruxism. The learners are in a community emergency department with intensive care capabilities and usual available consultants.* | | |
| **Confederates (e.g., standardized participants) and their roles in the room at case start** | *Nurse – present at the start of the case. May be played by anyone with basic medical knowledge (trained simulation technicians, EMT, medical student, MD).*   - *Verbal Script – “Hey Doc, can you come look at this guy? EMS just dropped him off for presumed intoxication.” If asked – “ They found him at a dance club. He was alone and only had an ID on him. They don’t think he fell or anything like that, but I don’t really know anything else about him and the paramedics already left. “* | | |
| **HPI** | *Patient unable to give HPI due to altered mental status. All history per initial scripting by RN.* | | |
| **Past Medical/Surgical History** | **Medications** | **Allergies** | **Family History** |
| Unknown | Unknown | Unknown | Unknown |
| **Physical Examination** | | | |
| **General** | *Pt is lethargic. He mumbles in response to questions but is not coherent* | | |
| **HEENT** | *Normocephalic, no evidence of trauma*  *Pupils 7mm, equal round and reactive to light. No nystagmus*  *Oral mucosa pink, dry, bruxism* | | |
| **Neck** | *Supple, trachea midline* | | |
| **Lungs** | *Tachypneic. Clear to auscultation bilaterally, no wheezes, rales, or rhonchi* | | |
| **Cardiovascular** | *Tachycardic, regular. No murmur, no rub, no gallop. 2+ pulses in all extremities, which are warm and well perfused, without edema* | | |
| **Abdomen** | *Soft, nondistended, nontender to palpation* | | |
| **Neurological** | *Oriented to name only, localizes pain, eye opening on occasion and to pain, no clonus, no rigidity. Moving all extremities purposefully.* | | |
| **Skin** | *Slightly diaphoretic, warm. No ecchymosis/petechia/purpura* | | |
| **GU** | *Normal external genitalia, no lesions, nontender, no fullness* | | |
| **Psychiatric** | *Mumbling incoherently. Unable to further evaluate.* | | |

| Instructor Notes - Changes and CASE Branch Points | | |
| --- | --- | --- |
| **Intervention / Time point** | **Change in Case** | **Additional Information** |
| At onset – if team attempts to intubate patient based on initial assessment | The patient should perform purposeful movements, occasionally open eyes to demonstrate GCS >8. | *RN may also prompt– “I think his GCS is okay. He’s trying to swat at you. Why are we intubating again?”* |
| 3 minutes into the case – if team has not recognized hyperthermia and obtained a core temperature or is pursuing sepsis evaluation only | RN will alert the provider*: “Doctor, the patient feels really hot to touch.* *I don’t think I trust that oral temperature probe. He was biting down.”* | No consequence such as arrest, death as this would not allow for the time needed to work through the hyperthermia algorithm. Requiring learners to manage a significant sequalae at this time point will delay progression of the case. |
| 4 minutes into case -If team has not performed a physical exam to demonstrate cause of hyperthermia and delineate sympathomimetic toxidrome | *RN will prompt, “What do you think this is? Is this something he needs like a specific treatment or antidote for? How would we know which it is?* |  |
| 5 minutes | If at least 1 cooling measure not initiated the patient will have a ventricular fibrillation arrest |  |
| If ventricular fibrillation arrest | Will respond to one round of CPR and one defibrillation with stable vitals |  |
| 10 minutes | If chilled IV fluids, ice pack placement, and evaporative cooling are not initiated the RN will prompt | *“I don’t think this is working. Is there something else we can try? I remember something with a [fan, ice packs, cooled fluids]”* |
| After 3 methods (IVF, ice pack placement, mist+fan) of cooling tried | If team has not asked for repeat temperature or placed a continuous monitoring method (foley, rectal) then the RN will prompt for a recheck. | *“How long do we need to keep this fan going? Do we even know how cold he is right now?”* |
| Team recognizes insufficient response to conservative cooling measures; temperature is still 40.5C | Team should initiate whole body packing. | If your institution has specific devices like the CoolGuard system or other central venous method of cooling, specialized mattresses, etc., they will be unavailable. |
| If team does not know of whole body packing techniques or can’t obtain equipment | RN can prompt *– “I had one of these. You can actually put them in a body bag. I think the toxicology team left some instructions on how to do that.”* Hands cue card. See Appendix E. |  |
| 1 minute after ice water whole body packing is initiated | If team has not rechecked temperature the patient will begin shivering. | *RN – “He’s shivering. Do you think he is cool enough yet?”* |
| Temperature check after whole body packing (if team progresses to this technique in timely fashion) | The temperature will be 38.7C and the patient will start having a generalized tonic clonic seizure. |  |
| 15 minutes into the case | If the team has not attempted whole boy packing the patient should begin seizure activity to allow the case to progress in a timely fashion | *RN should prompt the patient via predetermined physical cue (tapping shoulder) or by stating “He looks more out of it. I think he is seizing.”* |
| Onset of tonic clonic seizure | Team should remove the patient from packing method *if* it was performed and initiate treatment for seizure activity. The patient will not respond to usual treatment (benzodiazepines or antiepileptics) |  |
| During seizure activity, if the team attempts to intubate | RN should delay – *“We don’t just intubate all seizures. Shouldn’t we try and treat the seizure first?”* |  |
| 1 minute after seizure onset | If no hypertonic saline or sodium bicarbonate given, labs can be given. |  |
| Hypertonic saline infusion started | Seizure activity will terminate |  |
| If whole body packing was **NOT** initiated **AND** after management of hyponatremia induced seizure activity | No consequence. The repeat temperature, if obtained, will be 38.7. | Allowing adequate response to cooling allows the team to appropriately disposition the patient to the MICU and completion of the case. Cooling methods would have to be discussed in the debrief. |
| If learners attempt invasive techniques such as gastric lavage or peritoneal lavage | RN should NOT allow | *“I am sure there are other things you can do besides that that aren’t so invasive.”* |

**Ideal Scenario Flow**

Before the learners enter the room they receive an introduction to the case from the nurse. Learners should assign a leader for the case. The team should ask the nurse at the bedside for a brief history as she was there to receive emergency medical services personnel (EMS). The nurse will inform them that EMS was called by a bouncer at a local dance club after he found the patient to be confused. No one was with him to give any history and all they had was a wallet and identification. Learners should immediately ask for a full set of vitals, place the patient on the monitor, and obtain a point of care glucose. The patient will not be cooperative for an adequate oral temperature due to MDMA induced bruxism and mild agitation. The team should note this, place restraints, mild sedation with benzodiazepines, and request a core temperature. Once hyperthermia is recognized the team should initiate continuous temperature monitoring via either a bladder or rectal probe and immediately begin cooling measures including chilled IV fluids, strategic ice pack placement, evaporative cooling with fan + mist, sponge, or equivalent. Given tachycardia they should also initiate IV fluids and obtain an ECG which will show sinus tachycardia. While this is ongoing learners should be concurrently performing a physical exam to delineate the etiologies of the patients altered mental status and delirium. The patient is diaphoretic with mydriasis, without clonus or rigidity. This should key learners into a sympathomimetic toxidrome. The team should note that appropriate cooling is not occurring with conservative measures (chilled IV fluids, strategic ice pack placement, and evaporative cooling) at which point the team should progress to whole body ice packing. Once the patient is appropriately cooled he will have a generalized tonic clonic seizure that will not respond to benzodiazepines or antiepileptic medications. Given the history of the patient being at dance club, bruxism, and sympathomimetic toxidrome this should key learners into possible MDMA ingestion which is known to induce hyponatremia due to an SIADH like syndrome. If the team does not recognize hyponatremia as a potential cause of refractory seizures the nurse will give the labs, prompting treatment for hyponatremia induced seizure. The learners should give hypertonic saline or sodium bicarbonate and the seizure will resolve at this time. The team should obtain a CT scan of the brain now that the patient is stabilized and appropriately cooled. After CT scan the team should admit the patient to the intensive care unit. If toxicology or poison control is called, they will be unavailable until the patient is completely managed. At that time, they will agree with care. The team should consider consulting renal for the concomitant rhabdomyolysis with renal injury as well as need for judicious sodium repletion in the setting of severe hyponatremia. Renal will recommend holding further fluid administration for now and they will see the patient in the ICU.

Potential Branch Points

- If the team does not get a set of vitals and a core temperature the RN will prompt
- When the team has obtained a core temperature and recognized hyperthermia they should initiate cooling measures. If this is not done by at least five minutes in the patient will have a ventricular fibrillation arrest which will resolve after 1 round of CPR and defibrillation.
- If the team fails to recognize an inadequate response to cooling and initiate whole body packing, the patient will become less responsive and more tachycardic. Regardless of cooling measures initiated the patient will have a seizure at 15 minutes into the case.
- If team does not check the patient’s temperature after appropriately cooled he will have severe shivering and if not done by 15 minutes the patient will seize whether in or out of the bag.
- If CT imaging is performed prior to cooling and/or seizure management the patient will have a ventricular fibrillation arrest. The patient will obtain ROSC with at least one round of CPR and a defibrillation. Vitals will be stable.

**Anticipated Management Mistakes**

1. Uncertainty about cooling techniques: While all groups of residents were aware of conservative cooling measures (Ice packs, mist+fan, chilled IVF), many of our learners were unfamiliar with methods for whole body packing and did not attempt this prior to case termination. This had to be discussed as an option in debrief. We created specific debriefing materials to cover this information (Appendix H).
2. Attempt to use invasive techniques such as gastric lavage or peritoneal lavage: While only occurring once, one resident requested materials for peritoneal lavage. We simply had the RN direct the learner away from this option by insisting there were less invasive measures.
3. Failure to place continuous temperature monitoring: Half of the teams in our scenario did not place a form of continuous monitoring, requiring them to ask for frequent temperature rechecks.
4. Uncertainty about dosing of hypertonic saline: More than half of our groups did not know how to dose hypertonic saline for hyponatremia. In our case we allowed learners to contact pharmacy for assistance. Pharmacy, voiced by the facilitator, would then give the appropriate dosing.
