# Supplementary material for: Sympathomimetic-Induced Hyperthermia and Hyponatremia: A Simulation Case for Emergency Medicine Residents
Source: MedEdPORTAL. 2021 Jan 29;17:11092. doi: 10.15766/mep_2374-8265.11092 (PMC7845472; doi:10.15766/mep_2374-8265.11092)
Supplement: Supplementary file 1 — Simulation Case Template.docxAlternate Simulation Case Template.docxEquipment List.docxLaboratory Results.docxBody Bag Cue Card.docxResident Questionnaire.docxCritical Action Checklist.docxBackground Info for Debrief.docx [file mep_2374-8265.11092-s001.zip › B. Alternate Simulation Case Template.docx]

**Appendix B: Alternate Simulation Case Template**

| Title | MDMA/Sympathomimetic Toxicity |  | Learners are Expected to demonstrate the following: |
| --- | --- | --- | --- |
| Authors | Elizabeth Black, MD  Ashlea Winfield, MD |  | ***Clinical Objectives:***   1. Demonstrate a linear approach to evaluating the patient with altered mental status 2. Identify hyperthermia and the underlying toxidrome utilizing physical exam and history 3. Initiate cooling methods for hyperthermia: cooled IV fluids, ice pack placement, methods of evaporative cooling such as fan + tepid water mist or sponge, and whole body packing 4. Manage seizure activity due to MDMA induced hyponatremia with hypertonic saline or sodium bicarbonate |

| Learner Group | | Description of Environment |
| --- | --- | --- |
| Names | Specialty | A 23 yo man is BIB EMS. EMS was called by bouncer or an altered patient found at a local dance club. Patient is altered and cannot provide much of a history. No one was with him. He has an ID with his name, Alexander Martel and age. |
| EM Residents | EM |  |

| Summary of what happens with the patient: |
| --- |
| **Flow**:  Pt arrives via EMS for AMS. Pt is altered and cannot provide much of a history. No one was with him. He is acutely intoxicated from an unknown sympathomimetic substance (MDMA). As a result of this ingestion the patient will be lethargic, hyperthermic, and hyponatremic. The team should then initiate cooling methods. The patient will not appropriately cool until whole body packing is performed. After repeat temperature check, the patient will have a seizure secondary to his hyponatremia. It will not resolve until the team gives hypertonic saline or sodium bicarbonate. If the team fails to initiate whole body packing in a timely fashion (by 15 minutes) the patient will seize. After the seizure, *regardless of cooling measures implemented* the patient’s hyperthermia will resolve (teaching point). Once this is done the team should call appropriate consultants (consider Renal for hyponatremia with rhabdomyolysis, Toxicology, ICU for final disposition). |

| **Age** | 23 | **Allergies** | Unknown | **Social** | Unknown |
| --- | --- | --- | --- | --- | --- |

| **Gender** | M | **Ethnicity** | Caucasian | **Diagnosis** | Sympathomimetic /MDMA tox |
| --- | --- | --- | --- | --- | --- |
| **Ht/Wt** | 5’10” / 75 kg | **Imaging** | CXR – below, CTH wnl | **Medications** | Unk |
| **Labs** | Attached | **EKG** | Sinus Tachycardia | **Other** | None |

| Report given to the learners at the start of the scenario (include confidentiality, fiction contract, who they are): |
| --- |
| An approximately 20 year old man is BIB EMS.. |

| Time | Vital Signs | Patient State/Scripting | Expected Learner Action |
| --- | --- | --- | --- |
| Stage 1  Initial Evaluation and Recognition of Hyperthermia | Temp 39.1C Oral  40.5 core *have them request core | GEN: lethargic, mumbles, not coherent.  Head: Normocephalic, atraumatic.  Pupils: 7mm, EERL. No nystagmus.  EENT: Dry oral mucosa, bruxism  Resp: CTA bilaterally  MSK: No deformity  Skin: diaphoretic, warm  Neuro: Oriented to name only, localizes pain, eye opening to pain, no clonus  Accucheck- 137 | Obtain history from RN, vitals, place patient on monitor  Accucheck  IV Access  Send labs   - CBC, CMP, Tox Panel, UA, CK, Coags, Cultures   ECG |
|  | HR: 155 |  |  |
|  | RR: 20 |  |  |
|  | BP: 105/66 |  |  |
|  | SpO_2_: 100% |  |  |
| Stage 2  Cooling | Temp: Cooling🡪 40.5 🡪 | Pt physical state unchanged  Team should initiate cooling   - Cold IVF, Ice Packs, Mist + Fan will only lower to 40.5 🡪 Proceed to whole body packing - **Once team asks for whole body packing 🡪 Next Stage** | Initiate Cooling   - Cold IVF, Ice packs, Mist+Fan - Place bladder probe/rectal probe |
|  | BP: 106/80 |  |  |
|  | HR: 138 |  |  |
|  | RR: Unchanged |  |  |
|  | SpO2: Unchanged |  |  |
| Stage 3  Seizure Management | Temp: 38.7C Core | While in the bag and after temperature recheck the patient begins seizing, team should remove.  *RN may prompt* – “are we going to resuscitate him in this bag?”  **Labs back here if learners not concerned for hyponatremia*****  Team should manage seizure   - Benzodiazepines or antiepileptics – Will not respond - Hypertonic Saline bolus/Sodium Bicarbonate   Poison Control/Tox Can Call Back 🡪 Will agree with care and ICU admit.  ICU accepts without issue🡪 END OF CASE  If called Renal will see promptly and discuss with ICU | Initiate whole body packing – if they don’t know how to do this *RN may give cue card*  Manage Rhabdomyolysis: Consult Renal to discuss need for gentle sodium repletion in the setting of rhabdomyolysis  CTH – unremarkable. Should be obtained after cooled and seizure managed. |
|  | HR: 160🡪 115 after seizure |  |  |
|  | RR: Unchanged |  |  |
|  | BP: 200/101🡪123/78 after seizure |  |  |
|  | SpO_2_: 94% |  |  |

*******Labs to be printed and cut into respective pieces to be handed out during case at discretion of facilitator********

| **WBC** | 16 (4-10 x 10^9^/L) |
| --- | --- |
| **HGB** | 13.7 (13-17 g/dL) |
| **HCT** | 38.1 (40%-52%) |
| **PLT** | 297 (15-400 x 10^9^/L) |

| **Na** | 115 (135-145 mmol/L) |
| --- | --- |
| **K** | 4.3 (3.505 mmol/L) |
| **Cl** | 98 (95-105 mmol/L) |
| **HCO3** | 22 (18-22 mmol/L |
| **BUN** | 26 (8-21 mg/dL) |
| **Cr** | 1.4 (.8-1.3 mg/dL) |
| **Glucose** | 113 (65-110 mg/dL) |
| **AST** | 1,572 (5-30 U/L) |
| **ALT** | 3,062 (5-30 U/L) |

| **Post Seizure VBG** | |
| --- | --- |
| **pH** | 7.10 (7.31-7.41) |
| **pO2** | 32 (30-49 mmHg) |
| **pCO2** | 70 (41-51 mmHg) |
| **Lactate** | 8 (.5-1 mmol/L) |

| **PT** | 12 (11-14 sec) |
| --- | --- |
| **PTT** | 26 (20-40 sec) |
| **INR** | .9 (.9-1.2 |

| **Creatine Kinase** | 7,204 (20-250 U/L) |
| --- | --- |

| **Troponin** | .012 (0-.04 ng/mL) |
| --- | --- |
