# Supplementary material for: Sympathomimetic-Induced Hyperthermia and Hyponatremia: A Simulation Case for Emergency Medicine Residents
Source: MedEdPORTAL. 2021 Jan 29;17:11092. doi: 10.15766/mep_2374-8265.11092 (PMC7845472; doi:10.15766/mep_2374-8265.11092)
Supplement: Supplementary file 1 — Simulation Case Template.docxAlternate Simulation Case Template.docxEquipment List.docxLaboratory Results.docxBody Bag Cue Card.docxResident Questionnaire.docxCritical Action Checklist.docxBackground Info for Debrief.docx [file mep_2374-8265.11092-s001.zip › C. Equipment List.docx]

Appendix C: Equipment List

- Simulated Patient – Live Actor
  - Clothing: Shirtless, pants or shorts okay.
  - Appearance: sweaty, can simulate by spraying with water bottle prior to participant arrival
  - No initial IV in place
- Restraints: soft preferred as less technical difficult place, less likely cause injury to actor. Team can verbalize they would like leather restraints, but use these instead.
- Saline lock x 2 taped to skin as a method of simulating IV
- Hyperthermia Management
  - Ice packs
  - Body Bag x 2
  - Spray bottle for or sponge for evaporative cooling
  - Fan if available
- Medications and Infusions
  - Intravenous fluids: Lactated ringers and normal saline
  - Hypertonic Saline
  - Sodium Bicarbonate (crash cart 50mL vials)
  - Sedation medication vials: Haldol, lorazepam, midazolam, ketamine
- Miscellaneous
  - Gloves
  - Cardiac Monitor
