# Supplementary material for: Sympathomimetic-Induced Hyperthermia and Hyponatremia: A Simulation Case for Emergency Medicine Residents
Source: MedEdPORTAL. 2021 Jan 29;17:11092. doi: 10.15766/mep_2374-8265.11092 (PMC7845472; doi:10.15766/mep_2374-8265.11092)
Supplement: Supplementary file 1 — Simulation Case Template.docxAlternate Simulation Case Template.docxEquipment List.docxLaboratory Results.docxBody Bag Cue Card.docxResident Questionnaire.docxCritical Action Checklist.docxBackground Info for Debrief.docx [file mep_2374-8265.11092-s001.zip › D. Laboratory Results.docx]

**Appendix D: Laboratory Results**

****Labs to be printed and cut into respective pieces to be handed out during case at discretion of facilitator****

| **WBC** | 16 (4-10 x 10^9^/L) |
| --- | --- |
| **HGB** | 13.7 (13-17 g/dL) |
| **HCT** | 38.1 (40%-52%) |
| **PLT** | 297 (15-400 x 10^9^/L) |

| **Na** | 115 (135-145 mmol/L) |
| --- | --- |
| **K** | 4.3 (3.505 mmol/L) |
| **Cl** | 98 (95-105 mmol/L) |
| **HCO3** | 22 (18-22 mmol/L |
| **BUN** | 26 (8-21 mg/dL) |
| **Cr** | 1.4 (.8-1.3 mg/dL) |
| **Glucose** | 113 (65-110 mg/dL) |
| **AST** | 1,572 (5-30 U/L) |
| **ALT** | 3,062 (5-30 U/L) |

| **Post Seizure VBG** | |
| --- | --- |
| **pH** | 7.10 (7.31-7.41) |
| **pO2** | 32 (30-49 mmHg) |
| **pCO2** | 70 (41-51 mmHg) |
| **Lactate** | 8 (.5-1 mmol/L) |

| **PT** | 12 (11-14 sec) |
| --- | --- |
| **PTT** | 26 (20-40 sec) |
| **INR** | .9 (.9-1.2 |

| **Creatine Kinase** | 7,204 (20-250 U/L) |
| --- | --- |

| **Troponin** | .012 (0-.04 ng/mL) |
| --- | --- |
