# Supplementary material for: Sympathomimetic-Induced Hyperthermia and Hyponatremia: A Simulation Case for Emergency Medicine Residents
Source: MedEdPORTAL. 2021 Jan 29;17:11092. doi: 10.15766/mep_2374-8265.11092 (PMC7845472; doi:10.15766/mep_2374-8265.11092)
Supplement: Supplementary file 1 — Simulation Case Template.docxAlternate Simulation Case Template.docxEquipment List.docxLaboratory Results.docxBody Bag Cue Card.docxResident Questionnaire.docxCritical Action Checklist.docxBackground Info for Debrief.docx [file mep_2374-8265.11092-s001.zip › E. Body Bag Cue Card.docx]

**Appendix E. – Cue Card for Body Bag Technique**

1. Open both body bags
2. Put body bag #1 into body bag #2 . **When open, both zippers should be at the patient’s feet.**
3. Place patient in body bag #1. Again zipper on the open bags should be at the patient’s feet.
4. Zip body bag #1, +/- strategic ice packs
   1. When you close the zipper, stop at the level of patient’s neck, leaving this area exposed
5. Fill body bag #2 with ice and water then partially zip in the same fashion as body bag #1
