# Supplementary material for: Sympathomimetic-Induced Hyperthermia and Hyponatremia: A Simulation Case for Emergency Medicine Residents
Source: MedEdPORTAL. 2021 Jan 29;17:11092. doi: 10.15766/mep_2374-8265.11092 (PMC7845472; doi:10.15766/mep_2374-8265.11092)
Supplement: Supplementary file 1 — Simulation Case Template.docxAlternate Simulation Case Template.docxEquipment List.docxLaboratory Results.docxBody Bag Cue Card.docxResident Questionnaire.docxCritical Action Checklist.docxBackground Info for Debrief.docx [file mep_2374-8265.11092-s001.zip › F. Resident Questionnaire.docx]

**Appendix F – Resident Survey**

The following questionnaire aims to assess participant attitudes towards the simulated case: Management of Acute MDMA (3,4-Methyl​enedioxy​methamphetamine) Intoxication. Participation in this survey is voluntary. Should you participate, your responses will remain anonymous.

1. PGY level
   1. PGY1
   2. PGY2
   3. PGY3
   4. PGY4
2. This case gave me a better approach to the differential diagnosis of a patient with hyperthermia?
   1. Yes
   2. No
   3. Unsure
3. My knowledge of cooling techniques for hyperthermia were improved with this case?
   1. Yes – continue to question 4 if you answer “Yes”
   2. No – proceed to question 5 if you answer “No”
   3. Unsure – proceed to question 5 if you answer “Unsure”
4. If you answered yes to question 3, which cooling techniques have you become more aware of? (answer all that apply).
   1. Cold IVF
   2. Fan + Mist
   3. Ice Pack Placement
   4. Immersion Techniques
5. My knowledge of the utility of benzodiazepines in sympathomimetic overdose was improved.
   1. Yes
   2. No
   3. Unsure
6. Prior to participation in this simulated scenario, which of the following side effects of sympathomimetic overdose were you aware of? (select all that apply)
   1. Myocardial infarction
   2. Aortic Dissection
   3. Intracranial Hemorrhage
   4. Prolonged psychologic symptoms
   5. SIADH like syndrome
   6. Cardiac dysrhythmias and/or sudden cardiac death
   7. Seizures
7. Do you feel your participation in this case will change your clinical practice regarding the management of sympathomimetic toxicity?
   1. Yes
   2. No
   3. Unsure
8. Additional comments ____________________________________________________________________ ______________________________________________________________________________________
