# Supplementary material for: Sympathomimetic-Induced Hyperthermia and Hyponatremia: A Simulation Case for Emergency Medicine Residents
Source: MedEdPORTAL. 2021 Jan 29;17:11092. doi: 10.15766/mep_2374-8265.11092 (PMC7845472; doi:10.15766/mep_2374-8265.11092)
Supplement: Supplementary file 1 — Simulation Case Template.docxAlternate Simulation Case Template.docxEquipment List.docxLaboratory Results.docxBody Bag Cue Card.docxResident Questionnaire.docxCritical Action Checklist.docxBackground Info for Debrief.docx [file mep_2374-8265.11092-s001.zip › G. Critical Action Checklist.docx]

**Appendix G: Critical Action Checklist**

| **Critical Action** | **Check If Performed** |
| --- | --- |
| Obtain a full set of vital signs |  |
| Obtain a core temperature, rectal or bladder probe |  |
| Obtain stat glucose in patient with altered mental status |  |
| Attempt to obtain prehospital history from RN |  |
| Verbalize diagnosis as sympathomimetic toxidrome |  |
| Order chemical and physical restraint due to agitation and to facilitate exam |  |
| Send CBC, CMP, Toxicology Panel, and creatine kinase (CK) |  |
| Initiate cooling with ALL of the following: cold IV fluids, evaporative (mist+fan or sponge+fan or wet blanket+fan) cooling, and ice packs |  |
| Recognize failure to adequately respond to conservative cooling measures and progress to whole body packing or other more invasive method of cooling |  |
| Discontinue cooling methods when the patient is appropriately cooled to approximately 102F to prevent overshoot hypothermia |  |
| Manage hyponatremic seizure with hypertonic saline bolus or sodium bicarbonate |  |
| Obtain a CT Brain for a patient with acute altered mental status and new onset seizure activity |  |
| Contact poison control or toxicology service |  |
| Disposition patient to Medical Intensive Care Unit |  |
