# Supplementary material for: Sympathomimetic-Induced Hyperthermia and Hyponatremia: A Simulation Case for Emergency Medicine Residents
Source: MedEdPORTAL. 2021 Jan 29;17:11092. doi: 10.15766/mep_2374-8265.11092 (PMC7845472; doi:10.15766/mep_2374-8265.11092)
Supplement: Supplementary file 1 — Simulation Case Template.docxAlternate Simulation Case Template.docxEquipment List.docxLaboratory Results.docxBody Bag Cue Card.docxResident Questionnaire.docxCritical Action Checklist.docxBackground Info for Debrief.docx [file mep_2374-8265.11092-s001.zip › H. Background Info for Debrief.docx]

**Appendix H: Sympathomimetic Toxicity and Cooling**

**Clinical Manifestations**

- The combination of hyperthermia and hyperadrenergic state can lead to end-organ damage: DIC, MI (vasospasm and well as increased atherosclerotic disease), rhabdomyolysis, renal injury, cerebrovascular ischemia and hemorrhage, QT prolongation and dysrhythmias, Aortic Dissection, hyponatremia, seizures
  - Target work up to evaluate for these injuries – Don’t forget CK for rhabdomyolysis, ECG, Trop
    - ECG changes: Most common ECG abnormality was prolonged QTc interval 🡪 risk for ventricular dysrhythmias.

**Management**: **The longer these patients remain hyperthermic 🡪 increased morbidity and mortality**

- **Benzodiazepines!**
  - Cornerstone of the treatment of hyperthermia and the sympathomimetic toxidrome is the control of psychomotor agitation and adrenergic stimulation. Must chemically control. **Be liberal with benzodiazepines**.
  - No single benzodiazepine has been found to be clinically more effective in this case
    - Midazolam or diazepam will provide the desired pharmacokinetics, rapid onset, and ability to titrate
    - There is no standard or formally recommended dosing. Consider escalating doses as needed and titrate to effect (decreased agitation).
- Cooling Methods
  - Cooling caveats
    - Because rectal temperature lags behind core (esophageal) temperature, evaporative cooling should be discontinued when rectal temperature reaches 39°C (102.2°F). Continued cooling beyond this temperature may lead to overshoot hypothermia.
  - **Cooled IVF** via peripheral or CVC
  - **Strategic Ice Packing**: Anterior aspect of neck (carotid/jugular vessels), axilla, groin
    - Cooling in these areas occurs despite cutaneous vasoconstriction because of direct conductive heat loss from blood within the vessel and across the vessel wall, subcutaneous tissue, and skin to ice.
  - Evaporative Cooling
    - In practice, evaporative cooling is very efficient
    - How - Undress the patient. Position a fan at the foot of the bed as close to the patient as possible. Then sponge or mist the patient's skin continuously with tepid water. It is important to keep as much of the body surface area as moist as possible and exposed to airflow

## Whole-Body Ice Packing

- - - Method: undress the patient and then cover the extremities and torso with crushed ice. A fan blown over the patient may increase cooling. Can usually be performed on an ED stretcher without additional equipment, but a body bag is ideal. If not available- plastic cloths or trash bags may be placed under the patient with the edges curled up
    - Difficulties: Monitoring can be difficult because of shivering and displacement of electrodes.
  - Complete Immersion
    - Complete submersion of the patient in a tub filled with chilled water and ice is effective though less practical.
    - **Immersion cooling is not recommended for patients with unstable cardiac rhythms or those at risk for the development of these rhythms. A significant change in cardiac rhythm might go undetected during the labor-intensive process of immersion cooling.**
  - Invasive Internal Techniques to be considered when above methods don’t work
    - Cold Gastric Lavage: This technique should be reserved for patients whose airway is protected
    - Cold Peritoneal Lavage
    - Cold Hemodialysis
    - Bladder Lavage
- Paralysis
  - In severe cases, paralysis with a nondepolarizing agent may be required to prevent further heat production (due to agitation or seizure). Care must be taken, because paralysis can mask seizure activity
  - Benzodiazepines should be given concurrently to treat toxidrome
  - No succinylcholine due to risk of hyperkalemia in rhabdomyolysis
- Management of hyponatremia induced seizures
  - Hypertonic Saline (3% saline)
    - Dose usually **2 ml/kg**. So you usually give a 100-150 ml bolus over anywhere from 10-20 minutes. May repeat x2 10 mins later if continued symptoms.
    - Downside is that it may not be readily available. As an alternative sodium bicarbonate is usually rapidly accessible from crash cart.
    - Be sure to repeat electrolytes
    - Note: You will see some slight variations in dosing and bolus time in the literature but most center on a 2ml/kg bolus, usually settling on 150ml of 3% hypertonic saline over 15 minutes with ability to repeat up to two additional times after approximately 10 minutes.
  - Sodium Bicarbonate
    - Comes in 50ml vial of 1mEq/mL
    - **Dose of two ampules (100 mL) over 5-10 minutes**
    - Contraindicated in metabolic alkalosis
    - Be sure to repeat electrolytes
