# Supplementary material for: Sympathomimetic-Induced Hyperthermia and Hyponatremia: A Simulation Case for Emergency Medicine Residents
Source: MedEdPORTAL. 2021 Jan 29;17:11092. doi: 10.15766/mep_2374-8265.11092 (PMC7845472; doi:10.15766/mep_2374-8265.11092)
Supplement: Supplementary file 1 — Simulation Case Template.docxAlternate Simulation Case Template.docxEquipment List.docxLaboratory Results.docxBody Bag Cue Card.docxResident Questionnaire.docxCritical Action Checklist.docxBackground Info for Debrief.docx [file mep_2374-8265.11092-s001.zip › _Educational_Summary_Report_11092.pdf]

MedEdPORTAL® The AAMC Journal of Teaching and Learning Resources

Original Publication Open Access

Sympathomimetic-Induced Hyperthermia and Hyponatremia: A Simulation Case for Emergency Medicine Residents

Ashlea Winfield, MD, MSPH*, Elizabeth Black, MD, Michelle Sergel, MD

*Corresponding author: awinfield@cookcountyhhs.org

Abstract

Introduction: MDMA (3,4-methylenedioxymethamphetamine) is a popular drug of abuse associated with a variety of clinical manifestations. There are a number of life-threatening sequelae, including, but not limited to, agitated delirium, cardiac dysrhythmias, and hyperthermia. Similar to other substances that cause sympathomimetic toxidromes, MDMA also induces a syndrome of inappropriate antidiuretic hormone secretion-like state resulting in hyponatremia. The management of hyperthermia is of particular importance, as time to correction, particularly at temperatures greater than 106 °F, is directly associated with increased risk of morbidity and mortality. **Methods:** We created a simulation-based intervention to address and improve clinical skills relating to the management of MDMA intoxication. The scenario used a simulated patient to teach emergency medicine residents how to properly diagnose sympathomimetic toxicity and manage resultant hyperthermia and hyponatremia with cooling measures and appropriate fluid administration. Learners participated in a debrief session and were given an anonymous survey to assess their perceived knowledge. The case was performed as part of monthly emergency medicine resident didactics. **Results:** Eighteen learners took part in the case, with a 100% response rate. All participants agreed that the scenario increased their knowledge of cooling methods in severe hyperthermia, particularly whole-body packing. Eighty-nine percent ($n = 16$) reported that the scenario changed their practice patterns. **Discussion:** This simulated scenario requires minimal resources and can be instituted with emergency medicine residents from all levels of training. The scenario achieved its primary goal of improving residents' perceived knowledge of cooling measures in severe hyperthermia.

Keywords:
Sympathomimetic Toxidrome, MDMA, 3,4-Methylenedioxymethamphetamine (MDMA), Hyperthermia, Symptomatic Hyponatremia, Emergency Medicine, Medical Toxicology, Simulation

Educational Objectives

By the end of this simulation, learners will be able to:

1. Demonstrate a linear approach to evaluating a patient with altered mental status.
2. Identify hyperthermia and the underlying toxidrome utilizing physical exam and history.
3. Initiate cooling methods for hyperthermia: cooled IV fluids, ice pack placement, methods of evaporative cooling such as fan + tepid water mist or sponge, and whole-body packing.
4. Manage seizure activity due to MDMA-induced hyponatremia with hypertonic saline or sodium bicarbonate.

Citation:
Winfield A, Black E, Sergel M. Sympathomimetic-induced hyperthermia and hyponatremia: a simulation case for emergency medicine residents. *MedEdPORTAL*. 2021;17:11092.
https://doi.org/10.15766/mep_2374-8265.11092

Introduction

MDMA (3,4-methylenedioxymethamphetamine) is a popular drug of abuse associated with a number of pathologic consequences. As MDMA is a methamphetamine derivative, clinical sequalae are consistent with other sympathomimetic toxidromes. There are a number of acute manifestations, including, but not limited to, cardiac dysrhythmias and ischemia, aortic dissections, rhabdomyolysis, and acute renal injury. Hyperthermia is a life-threatening effect of sympathomimetic toxidromes, and time to correction, particularly at temperatures greater than 106 °F, is directly associated with increased risk of morbidity and mortality.[1] Due to the time sensitivity in the management of severe hyperthermia, there is a need to ensure that emergency medicine physicians are familiar with the interventions needed to

Copyright © 2021 Winfield et al. This is an open-access publication distributed under the terms of the Creative Commons Attribution license.

treat these patients. An intensive simulation-based intervention could be crucial in addressing and improving clinical skills relating to the management of MDMA intoxication. Simulation is an excellent teaching modality for this case as it allows learners to walk through the hands-on skills needed for cooling, particularly techniques related to immersion and whole-body packing.

This case provides a unique contribution to the existing *MedEdPORTAL* literature as there are no cases related specifically to the sequalae of MDMA. While there is one problem-based learning (PBL) case[2] that describes the management of acute agitation and the differential diagnosis of chest pain in a patient with methamphetamine use, there are no simulated scenarios specifically related to this material. Outside of PBL-based cases, there are no simulation scenarios in *MedEdPORTAL* that address sympathomimetic toxidromes. This case also involves the management of hyponatremia from a syndrome of inappropriate antidiuretic hormone secretion-like (SIADH-like) phenomenon that is seen with MDMA. A previously published simulation curriculum for critical care fellows[3] covers seizures induced by symptomatic hyponatremia. While our case also covers the treatment of symptomatic hyponatremia, it does so in the context of a less commonly known complication of MDMA. It also expounds on the sequelae of MDMA abuse by allowing learners to manage more common symptoms, such mild agitation, hyperthermia, rhabdomyolysis, and renal injury.

Methods

Development

The simulation was created as a part of required emergency medicine resident didactics for PGY 1-PGY 3 residents at Cook County Health. Each class was split into two groups and assigned a 30-minute time slot in which to complete the case and a debriefing session. Groups were no larger than four participants. Learners needed to have basic knowledge of toxicologic emergencies. This scenario was appropriate for emergency medicine residents in all levels of training. No prerequisite lectures took place prior to the event as this would have alerted learners to the content of the case. Facilitators had to be at least senior-level emergency medicine residents.

Equipment/Environment

The case (Appendices A and B) was completed utilizing a simulated patient. It could alternatively be performed with a high-fidelity mannequin if preferred by facilitators. All standard items (as listed in Appendix C) were available. Moulage consisted of a shirtless male in shorts with simulated sweat, which could be achieved via spray bottle or other method of applying water to the skin (also detailed in Appendix C).

Personnel

Personnel consisted of a simulated patient, a nurse confederate, a technician, and a facilitator. The simulated patient, nurse, and technician could be any level of learner. The technician was trained in the operation of simulation software. The facilitator ideally was an emergency medicine attending physician who observed overall team performance, guided technicians on patient status changes in response to learner actions, and facilitated the debriefing session. The facilitator could also voice consultants or alert the team to the availability or lack thereof of consulting services. The nurse played a particularly important role in the case as they held the responsibility for maintaining the case's progression by giving clues as to appropriate management if learners were unsure of next steps. All personnel were required to have reviewed the case and debrief notes before to their arrival on the day of the simulation session. Prior to the start of the scenario, the facilitator discussed the overall flow of the case with all personnel. This discussion included reviewing physical exam findings, time points at which the nurse should give learners specific lab results (Appendix D) or certain prompts, and what signal the simulated patient would receive to indicate they should begin seizure activity.

Implementation

This simulated scenario involved emergency medicine residents at their monthly didactic sessions in a simulation center. Learners had been previously oriented to the simulation center and the use of simulated patients due to prior exposures in monthly resident conference. The setting was an emergency department. The case was performed in order to expose learners to hyperthermia, a less commonly seen presentation of sympathomimetic toxicity, and to give them needed exposure to efficiently cooling patients.

Prior to entering the room, learners were met outside by a confederate nurse who gave them a brief introduction: "EMS just dropped off this guy and left. They said he was acting strange, taking off his clothes at a local night club. No one else is with him and all we have is an ID. Can you take a look at him?"

Throughout the case, the nurse prompted learners to ensure appropriate progression (e.g., repeating temperature, escalating care, or removing the patient from the body bag) or to prevent an unnecessary procedure that would potentially delay the case (e.g., intubating immediately upon noticing seizure). We anticipated that learners would not have a strategy for whole-body packing or similar immersive techniques, so we created a cue card detailing how to complete the process (Appendix E)

Copyright © 2021 Winfield et al. This is an open-access publication distributed under the terms of the Creative Commons Attribution license.

that the nurse would hand out if they sensed that the team was unsure how to proceed. The nurse was also responsible for keeping track of time and ensuring the patient seized with at least 5 minutes remaining until the end of the case. This was done by having the nurse give a physical or verbal cue to the simulated patient such as “He looks a little more out of it. I think he is seizing,” or a predetermined signal such as a pat on the shoulder. This forced progression ensured learners were given a chance to manage the hyponatremia-induced seizure.

The facilitator and simulation technician were in a different room with a one-way mirror and audiovisual equipment to observe, communicate relevant information to learners, and voice consultants. The facilitator also guided vital sign changes based on the interventions performed. We found that the play of the case took at least 20 minutes to complete. The case is fully presented in two different formats in Appendices A and B.

Assessment

Assessment of the simulation was performed via an anonymous questionnaire (Appendix F) developed by two emergency medicine physicians with formal simulation training. This questionnaire was given exempt status (Cook County Health Institutional Review Board, #20-043X, April 14, 2020). The survey was designed to be a measure of participant attitudes towards perceived knowledge. Ideally, residents would have taken a pre- and postsurvey to assess knowledge; however, administering a survey prior to the case would have alerted learners to the content being covered in the simulation. Residents were asked about their knowledge in regard to the differential diagnosis of hyperthermia and causative toxidromes, clinical sequelae of sympathomimetic toxicity, and management of severe hyperthermia.

Debriefing

Debriefing sessions lasted approximately 10 minutes after completion of the case and were conducted primarily by the facilitator. At our institution, an emergency medicine physician assumed the role of the simulated patient and therefore was able to give feedback from that perspective. No checklists were completed during the scenario; instead, the facilitator took notes on the resident’s management of the case. The debrief was conducted as an open forum with a small component of advocacy and inquiry questioning to ensure core learning objectives were discussed. For the use of future facilitators, we have included a critical action checklist (Appendix G) and background information for the debriefing (Appendix H) in this publication.

Results

This simulation was run six times consecutively, with two to four learners participating at a time. It was performed on a single conference date, and therefore, participation was limited to residents who were present at didactics at that time. The case has not been reused because of the inability of convening groups due to social distancing measures during the COVID-19 pandemic. Case evaluations were overall positive. Eighteen learners took part in the case, with a 100% response rate. All 18 agreed that the scenario increased their knowledge of cooling methods in severe hyperthermia specifically regarding whole-body packing techniques. Most residents (>60% for all sequelae listed) were unaware that myocardial infarction, aortic dissection, intracranial hemorrhage, and an SIADH-like syndrome/hyponatremia were known sequelae of sympathomimetic toxicity. Sixteen (89%) reported that the scenario altered their future practice patterns. See the Table for a summary of questionnaire results.

Discussion

Hyperthermia is one of the most life-threatening acute consequences of MDMA intoxication, and failure to promptly recognize and treat it is directly related to increased morbidity and mortality. Careful cooling remains the mainstay of treatment, but many learners are not aware how to implement cooling methods, particularly whole-body packing techniques. Additionally, there are a number of clinical sequelae that should be investigated upon presentation, including renal injury, rhabdomyolysis, acidosis, and hyponatremia. Without knowledge of these effects it can be difficult to obtain a targeted workup. We feel that our case presents a unique addition to the *MedEdPORTAL* community. While there is already a PBL case in *MedEdPORTAL* involving the management of agitation and chest pain in methamphetamine use,[2] ours is

Table. Summary of Resident Questionnaire Results

Statement	Answer (%) Yes	No
Felt the case gave a better approach to the differential diagnosis of hyperthermia.	22	78
Knowledge of cooling techniques in hyperthermia improved.	100	0
Knowledge of the use of cold intravenous fluids improved.	19	81
Knowledge of the use of mist + fan for evaporative cooling improved.	63	27
Knowledge of ideal ice pack placement in hyperthermia improved.	19	81
Knowledge of immersive techniques improved.	89	11
Knowledge of the utility of benzodiazepines in sympathomimetic toxicity improved.	39	61
Felt that participation in this case will change future clinical practice.	89	11

Copyright © 2021 Winfield et al. This is an open-access publication distributed under the terms of the Creative Commons Attribution license.

the only simulation scenario involving a sympathomimetic toxidrome and the only educational material specific to MDMA.

This case is appropriate for all levels of emergency medicine resident learners. Overall, residents felt that their knowledge of cooling methods in hyperthermia improved and that their knowledge of the clinical consequences of MDMA and related ingestions increased. While we found that learners were aware of basic cooling measures, none of the groups had a clear strategy for whole-body packing techniques. We exposed learners to an easily accomplishable method of cooling involving low-cost materials that can be found at any hospital. Cooling techniques for sympathomimetic-induced hyperthermia can also be used in other toxidromes, as well as in environmental causes such as heatstroke, giving learners a broader application for the techniques learned in this simulation.

We performed this case with a simulated patient to allow participants to more easily see changes in mental status and to better demonstrate seizure activity. The case could also be done with a high-fidelity simulator with the capability of mimicking seizure activity or with a low-fidelity simulator relying on verbal cues from the confederate or facilitator. The case does not have to be performed in a simulation lab, and we believe it can be performed as part of an in situ simulation. In situ simulation allows examination of possible system issues and can help ascertain accessibility and availability of the supplies needed for cooling. While preparing for this case, we had multiple communications with different departments to ascertain where we could obtain an industrial fan or how to contact the morgue for body bags. This helped to delineate potential systems issues.

There were several challenges encountered while running the scenario. Time was the biggest limiting factor. At our institution, we were allotted 30 minutes not only to complete the scenario and debrief but also to allow the residents to transition to the simulation lab from other lectures. While all learning points were discussed in the debrief, there was not as much time for learners to freely ask questions, which limited our capability to appropriately utilize the advocacy-inquiry method. In order to protect time for the debrief, the seizure activity began by 15 minutes into the case to ensure we had a chance to discuss seizure management and symptomatic hyponatremia. Ideally, we would advise allowing for at least an additional 10 minutes in order to give learners the opportunity to perform whole-body packing and to allow a more in-depth discussion during the debriefing session.

Another limitation of our study is that our primary outcome is focused on resident perception rather than actual measures of knowledge. Ideally, we would obtain a pre- and postsurvey, but we felt this was not appropriate as it would have alerted learners to the clinical concepts being covered and would not allow them to work through an approach to the undifferentiated patient.

One issue with this simulation is that there are a variety of cooling methods available for patients who are refractory to more conservative measures. Some institutions utilize patented central venous catheter systems for internal cooling, mattresses, or other specialized devices to allow for temperature management. Our case does not permit learners to use these items as they are not widely available in community centers. We believe conducting the case without the more advanced methods teaches learners to troubleshoot when these rarer items are not available. This is not an issue at our institution because we do not carry any of the aforementioned equipment. For institutions that do have cooling catheters and more advanced techniques of cooling, the case could also accompany a breakout skills session or side trainer where a resident could place the catheter while the clinical scenario progresses. If the catheter is introduced in the case, it is advisable to allot more time to allow for review of the procedural skill.

In summary, we feel this simulation exposes learners to a less common manifestation of sympathomimetic toxicity that carries significant morbidity and mortality if not rapidly controlled. The cooling techniques taught in this case not only can be applied broadly to other causes of hyperthermia but also can be utilized at any health care facility. We will continue to use this simulation as part of our longitudinal simulation curriculum for future iterations.

Appendices

A. Simulation Case Template.docx

B. Alternate Simulation Case Template.docx

C. Equipment List.docx

D. Laboratory Results.docx

E. Body Bag Cue Card.docx

F. Resident Questionnaire.docx

G. Critical Action Checklist.docx

H. Background Info for Debrief.docx

All appendices are peer reviewed as integral parts of the Original Publication.

Copyright © 2021 Winfield et al. This is an open-access publication distributed under the terms of the Creative Commons Attribution license.

Ashlea Winfield, MD, MSPH: Simulation Fellow, Department of Emergency Medicine, Cook County Health; ORCID: https://orcid.org/0000-0001-6787-7980

Elizabeth Black, MD: Toxicology Fellow, Department of Emergency Medicine, Cook County Health

Michelle Sergel, MD: Director of Cook County Simulation Center, Department of Emergency Medicine, Cook County Health; Co-Executive Director, Rush Center for Clinical Skills and Simulation

Disclosures
None to report.

Funding/Support
None to report.

Ethical Approval
The Cook County Health Institutional Review Board approved this study.

References

1. Rao RB, Hoffman RS, Erickson TB. Cocaine and other sympathomimetics. In: Walls RM, Hockberger RS, Gausche-Hill M, et al., eds. *Rosen's Emergency Medicine: Concepts and Clinical Practice*. 9th ed. Elsevier; 2018:1895-1903.
2. Barone E, Sattar SP, Huggett K. Two problem-based learning cases: methamphetamine. *MedEdPORTAL*. 2011;7:8384. https://doi.org/10.15766/mep_2374-8265.8384
3. Keller JM, Steinbach TC, Adamson R, et al. ICU emergencies simulation curriculum for critical care fellows: neurologic emergencies. *MedEdPORTAL*. 2019;15:10813. https://doi.org/10.15766/mep_2374-8265.10813

Received: June 9, 2020
Accepted: October 31, 2020
Published: January 29, 2021

Copyright © 2021 Winfield et al. This is an open-access publication distributed under the terms of the Creative Commons Attribution license.
